# Comparative Size and Shape of the Thyroid Foramen in the Male and Female Innominatum

**Published:** 1850-10

**Authors:** 


### RECORD OF MEDICAL SCIENCE.

### ANATOMY AND PHYSIOLOGY.

*Comparative size and shape of the Thyroid Foramen in the Male and Female Innominatum.* Dr. NEILL called the attention of the Fellows of the College to the comparative size and shape of the Thyroid Foramen in the Male and Female Innominatum.

He believed that many teachers of Anatomy and of Obstetrics were in error upon this subject, while others had failed to point out the difference between the male and female pelvis in this respect; that this was the case especially in this city, and perhaps in this country generally.

He had learned that Dr. Wistar and Dr. James taught that in the *female* the foramen was *oval*, and that in the *male* it was *triangular*, although there was no statement upon the subject in the old edition of Wistar's Anatomy which he had examined, nor in the more recent edition known as Pancoast's Wistar. Dr. Horner also stated in his work, that "in the male it is triangular, in the female, rather oval." On the other hand, Meckel, Cloquet, Cruveilhier, Harrison and Quain and Sharpey make a statement precisely the reverse of this.

The lecturers upon obstetrics with whom he had conversed, either teach the former view, or are silent upon the subject. Denman, Baudeloque, and Maygrier say nothing. Neither do Monroe or Cheselden, both high authority on the bones, nor Winslow, Bell, Bartholin, &c.

Sœmmerring says "the foramen is elliptical in children, and triangular in adults." Wilson and Von Behr say it is triangular in women.

In order to satisfy his own mind on the subject, Dr. Neill had, up to this period, examined thirty-two skeletons, and the result was so contrary to the view which he, and perhaps most of the Fellows had been taught, that he had thought it worth while to prepare a chart, exhibiting diagrams of the male innominata in one column, and those of the female in another, to show at a glance, the distinctive difference.

He believed from an inspection of this, every one would be convinced that the foramen in the *male* is *oval*, while in the *female* it is *triangular*.

It will also be observed that the male foramen is longer and narrower, and that the line representing the long axis is more vertical, and nearly parallel to the rami of the pubes and ischium; whereas, in the female the foramen is not only smaller and triangular, but that the apex of the triangle is downward, that its internal side is nearly parallel to the rami of the ischium and pubes; and that the base of the triangle is proportional to the chord of the arch of the pubes.

He remarked that the establishment of this fact, by investigation or by authority, would not interest the Fellows of the College in a practical point of view, its only value consisting in its affording another mark of distinction between the male and female skeletons.

*Trans. Col. Phys. Phil.*

